# Association of Glucagon-like Peptide-1 Receptor Agonists Versus Dipeptidyl Peptidase-4 Inhibitors with Tuberculosis and Mortality in Adults with Diabetes

**DOI:** 10.3390/ijms27209137

**Published:** 2026-10-15

**Authors:** Chun-Hsiang Hsu, Renin Chang, Chiu-Fan Chen, Kuo-An Chu, You-Cheng Jiang, Wei-Cheng Hong, I-Yuan Chen, Min-Hsi Lin

**Affiliations:** 1 Division of Chest Medicine, Department of Internal Medicine, Kaohsiung Veterans General Hospital, Kaohsiung 813414, Taiwan; benhsu740124@gmail.com (C.-H.H.);; 2 Doctoral Program of Clinical and Experimental Medicine, College of Medicine, National Sun Yat-sen University, Kaohsiung 804201, Taiwan; 3 Department of Medical Education and Research, Kaohsiung Veterans General Hospital, Kaohsiung 813414, Taiwan; 4 School of Medicine, College of Medicine, National Sun Yat-sen University, Kaohsiung 804201, Taiwan; 5 Cancer Center, Kaohsiung Veterans General Hospital, Kaohsiung 813414, Taiwan

**Keywords:** GLP-1 receptor agonist, DPP-4 inhibitor, diabetes, tuberculosis, mortality, retrospective cohort

## Abstract

Diabetes is associated with an increased risk of tuberculosis (TB), but whether the class of glucose-lowering therapy is associated with TB outcomes remains uncertain. We conducted a retrospective observational cohort study to examine the association between glucagon-like peptide-1 receptor agonist (GLP-1 RA) use versus dipeptidyl peptidase-4 inhibitor (DPP-4i) use and the risk of TB and mortality. We used de-identified records from the TriNetX Global Collaborative Network. Adults with diabetes who had a qualifying record for either a GLP-1 RA or a DPP-4i between 2016 and 2024 were eligible. Cohort entry was defined as the first qualifying drug-class record during this period. Patients with prior-year use of the comparator drug class, a history of TB, or death before cohort entry were excluded. Propensity score matching was performed at a 1:1 ratio. Outcomes assessed during days 1–365 included TB (ICD-10-CM A15–A19), the platform-defined death outcome, and a composite outcome of gastrointestinal and hepatobiliary diagnoses. After matching, 466,903 patients remained in each group. GLP-1 RA exposure was associated with fewer recorded TB events, deaths, and gastrointestinal/hepatobiliary events than DPP-4i exposure during the first year after cohort entry. TB was recorded in 155 GLP-1 RA users and 320 DPP-4i users (HR, 0.472; 95% CI, 0.390–0.572). The corresponding numbers for the platform-defined death outcome were 7575 and 14,572 (HR, 0.505; 95% CI, 0.492–0.520), and those for gastrointestinal/hepatobiliary diagnoses were 15,914 and 20,559 (HR, 0.750; 95% CI, 0.735–0.766). The analysis restricted to patients with type 2 diabetes (ICD-10-CM E11) yielded a similar association for TB. Residual imbalance in body mass index (BMI) and residual confounding, including confounding by indication, limit a causal interpretation.

## 1. Introduction

Tuberculosis (TB) remains one of the leading infectious causes of morbidity and mortality worldwide. Despite advances in diagnosis and treatment, TB continues to be prevalent, causing approximately 8.13 million new cases and 1.23 million deaths in 2024 [1]. Between 2015 and 2024, the net decline in TB incidence was 12.3%, far short of the World Health Organization’s End TB Strategy target of a 50% reduction in TB incidence by 2025. In addition, the number of TB deaths worldwide decreased by a net 29%, almost one-third of the way to the 75% reduction milestone set out in the World Health Organization’s End TB Strategy by 2025 [1]. Finding appropriate drug interventions to reduce the incidence and mortality of TB is an important goal at present.

In parallel, diabetes mellitus (DM) has emerged as a major global epidemic, with an estimated 589 million adults affected worldwide in 2025, and this number is predicted to rise to 853 million by 2050 [2]. The convergence of these two conditions poses a serious public health challenge, particularly in regions with high TB burden and increasing prevalence of diabetes. A growing body of evidence demonstrates that DM significantly increases the risk of developing active TB. Meta-analyses and large cohort studies have consistently shown that individuals with DM have a two- to three-fold higher risk of active TB compared with non-diabetic individuals [3,4,5,6,7,8,9]. The World Health Organization (WHO) estimated that in 2020, nearly 370,000 incident TB cases were attributable to DM, underscoring the contribution of metabolic disease to TB epidemiology [1]. According to a comprehensive systematic review and meta-analysis, the global pooled incidence of pulmonary TB (PTB) among patients with type 2 diabetes mellitus (T2DM) is estimated at 129.89 per 100,000 person-years (95% CI: 97.55–172.95), while the pooled prevalence stands at 511.19 per 100,000 (95% CI: 375.94–695.09) [10].

The association between diabetes and TB is not uniform across all diabetic patients; rather, it is strongly influenced by the degree of glycemic control. Poorly controlled diabetes, particularly with sustained hyperglycemia, is associated with higher TB incidence [11,12]. For instance, Lee et al. demonstrated in a large prospective cohort study that diabetic patients with elevated fasting glucose had approximately double the risk of TB compared with non-diabetic subjects, while those with well-controlled DM had no significant excess risk [11]. These findings suggest a dose–response relationship between hyperglycemia and TB susceptibility.

The biological mechanisms underpinning this association are increasingly understood. Chronic hyperglycemia impairs both innate and adaptive immune responses, including reducing macrophage function, impairing chemotaxis, causing defective antigen presentation, and diminishing T-helper 1 cytokine production [13,14]. These immune alterations compromise the host’s ability to contain *Mycobacterium tuberculosis* infection and may contribute to higher bacterial loads, delayed sputum conversion, and more severe radiographic disease in diabetic patients. In addition to increasing TB incidence, diabetes adversely affects TB treatment outcomes. Poor glycemic control has been associated with slower sputum conversion, higher rates of treatment failure and relapse, and increased mortality [12]. These observations highlight the dual role of glycemic control in both the prevention and treatment of TB among diabetic individuals.

Both glucagon-like peptide-1 receptor agonists (GLP-1 RAs) and dipeptidyl peptidase-4 inhibitors (DPP-4is) target the incretin pathway, yet they exhibit distinct pharmacological profiles and clinical trajectories. While DPP-4is prolong endogenous incretin activity at physiological levels with weight neutrality, GLP-1 RAs provide pharmacological receptor activation that yields superior glycemic efficacy, profound weight loss, and marked pleiotropic anti-inflammatory benefits [15,16,17]. The glucose-lowering effect of GLP-1 RAs is mediated through several well-characterized mechanisms. First, GLP-1 RAs enhance glucose-dependent insulin secretion from pancreatic β-cells, thereby improving postprandial and fasting glycemic control. Second, they suppress glucagon secretion from pancreatic α-cells, which reduces hepatic glucose output. In addition, GLP-1 RAs delay gastric emptying, leading to a slower rate of nutrient absorption. Finally, by acting on the central nervous system, particularly the hypothalamus, they reduce appetite and promote satiety [15,16]. Furthermore, landmark cardiovascular outcome trials and current international guidelines strongly endorse GLP-1 RAs over DPP-4is due to their proven capacity to reduce major adverse cardiovascular events (MACE), cardiorenal progression, and all-cause mortality in high-risk patients [17]. Nevertheless, real-world prescribing decisions are frequently constrained by drug costs, insurance coverage, supply shortages, and route of administration. Comparing GLP-1 RAs with DPP-4is as an active comparator class provides a critical biological and clinical framework to evaluate whether direct receptor stimulation translates into sustained survival and organ-protective advantages over indirect incretin preservation in routine clinical practice [17,18,19].

Gastrointestinal and hepatobiliary disorders represent clinically relevant safety outcomes of incretin-based therapies. GLP-1 RAs frequently cause upper and lower GI adverse events such as gastroesophageal reflux disease (GERD), nausea, and diarrhea through delayed gastric emptying [20,21,22]. Both GLP-1 RAs and DPP-4is have been linked to increased biliary risks, including cholecystitis and cholelithiasis, driven by impaired gallbladder motility and rapid weight loss [23,24,25]. These findings highlight the need for further large-scale real-world comparisons of the gastrointestinal and hepatobiliary safety profiles of GLP-1 RAs and DPP-4is.

We therefore compared recorded cases of TB, deaths, and gastrointestinal/hepatobiliary events after GLP-1 RA versus DPP-4i cohort entry. We also examined whether restricting eligibility to documented type 2 diabetes (ICD-10-CM E11) changed the findings. The study was observational and was not designed to establish treatment effects.

## 2. Results

### 2.1. Cohort Characteristics and Covariate Balance

**Figure 1:**
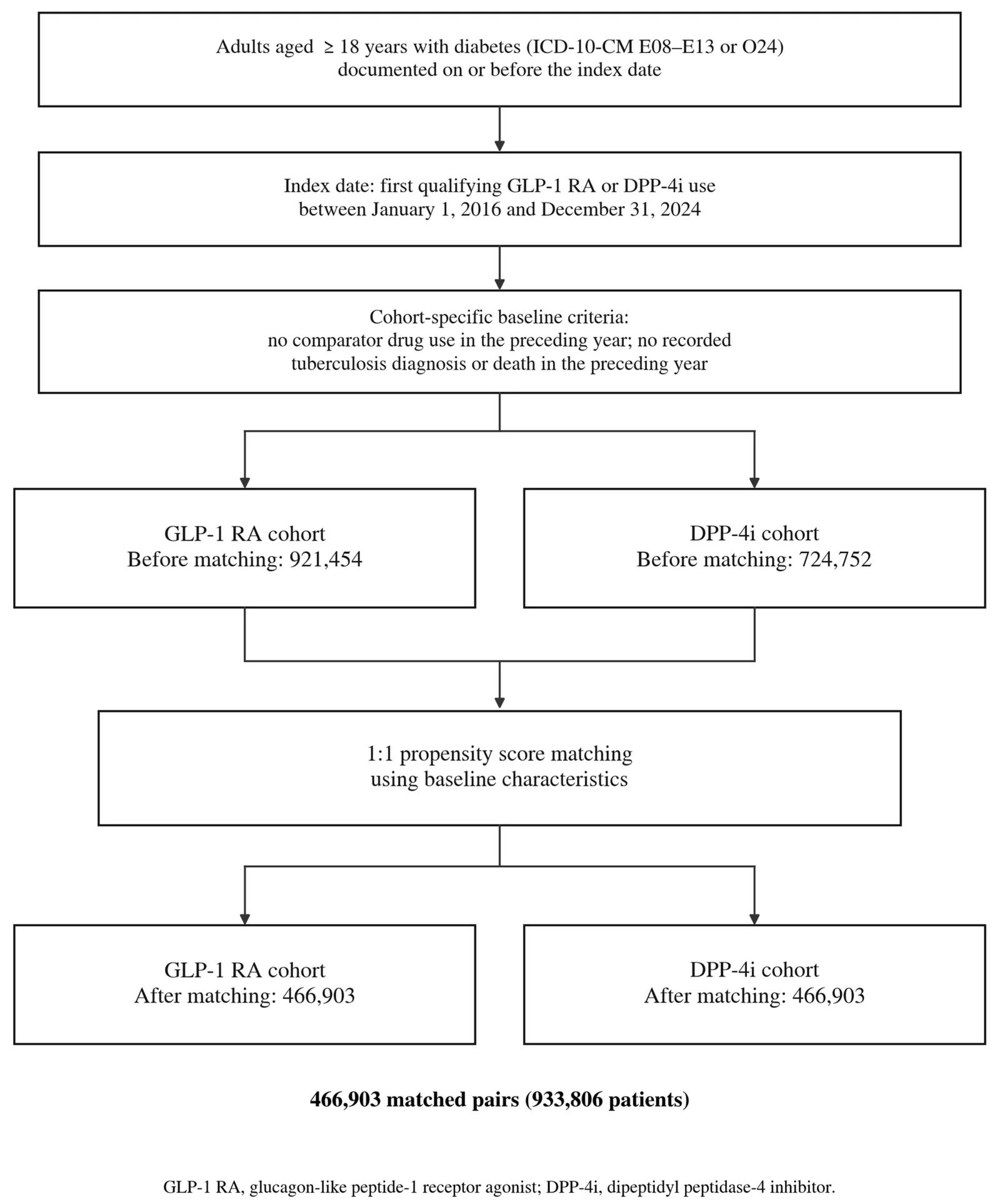
Study cohort selection and propensity score matching. The index date was the first qualifying GLP-1 RA or DPP-4i record during 2016–2024. The matched main analysis contained 466,903 pairs (933,806 patients).

The query identified 921,454 eligible GLP-1 RA users and 724,752 eligible DPP-4i users before matching. After 1:1 propensity score matching, there were 466,903 patients in each group, equivalent to 466,903 pairs or 933,806 individuals (Figure 1). Most reported covariates had absolute standardized mean differences below 0.1 after matching (Table 1). Body mass index remained imbalanced: 35.3 ± 7.8 kg/m^2^ in the GLP-1 RA group and 32.2 ± 7.5 kg/m^2^ in the DPP-4i group (absolute standardized mean difference, 0.406). Table 2 summarizes the absolute number of events, risk difference, and risk ratio.

### 2.2. Recorded Tuberculosis

**Figure 2:**
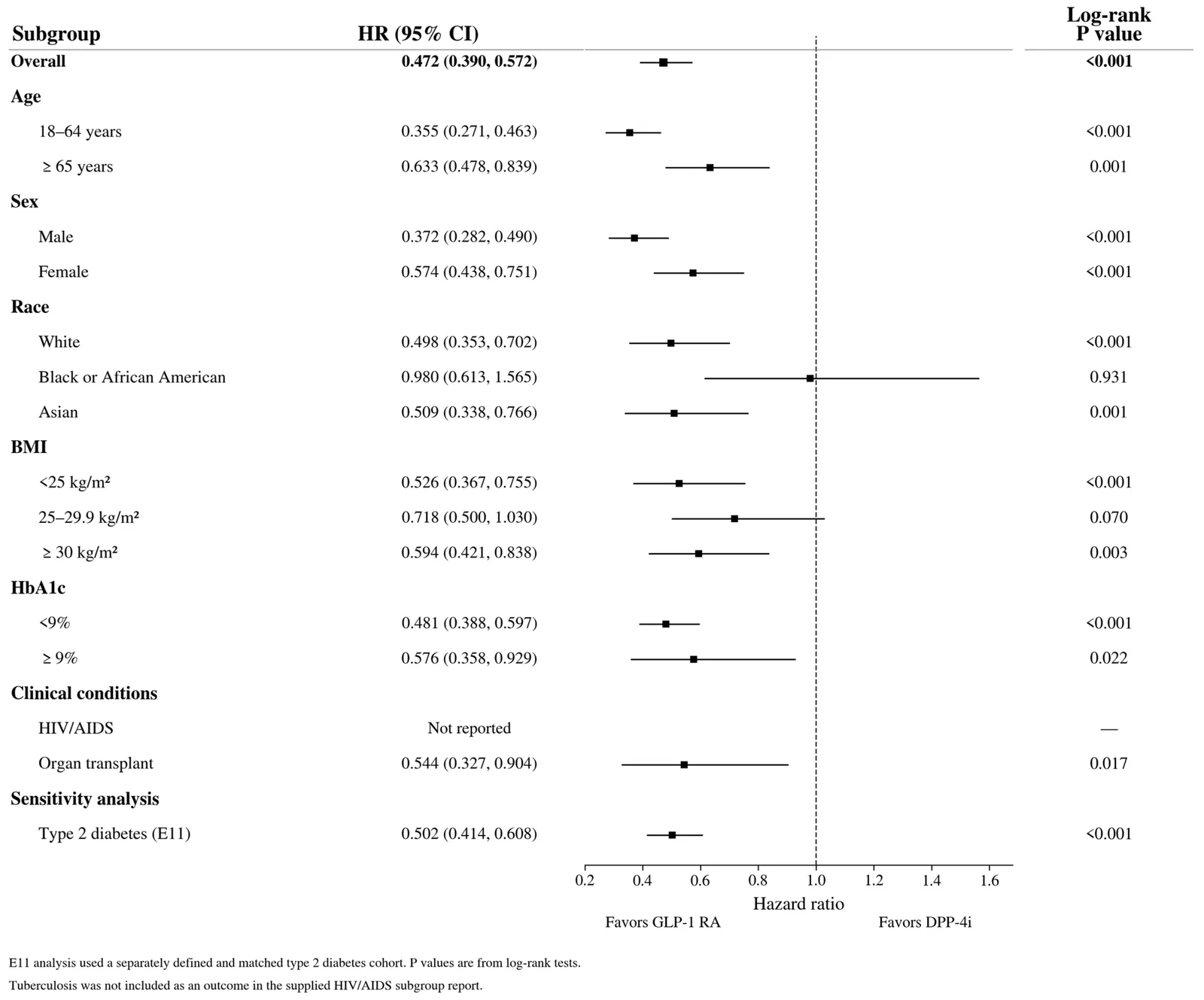
Exploratory subgroup comparisons and E11 sensitivity analysis for recorded tuberculosis. Squares indicate HRs, and lines show 95% confidence intervals. Separately matched subgroups do not provide formal tests of treatment-by-subgroup interaction. No TB estimate was reported for the HIV/AIDS stratum.

**Figure 3:**
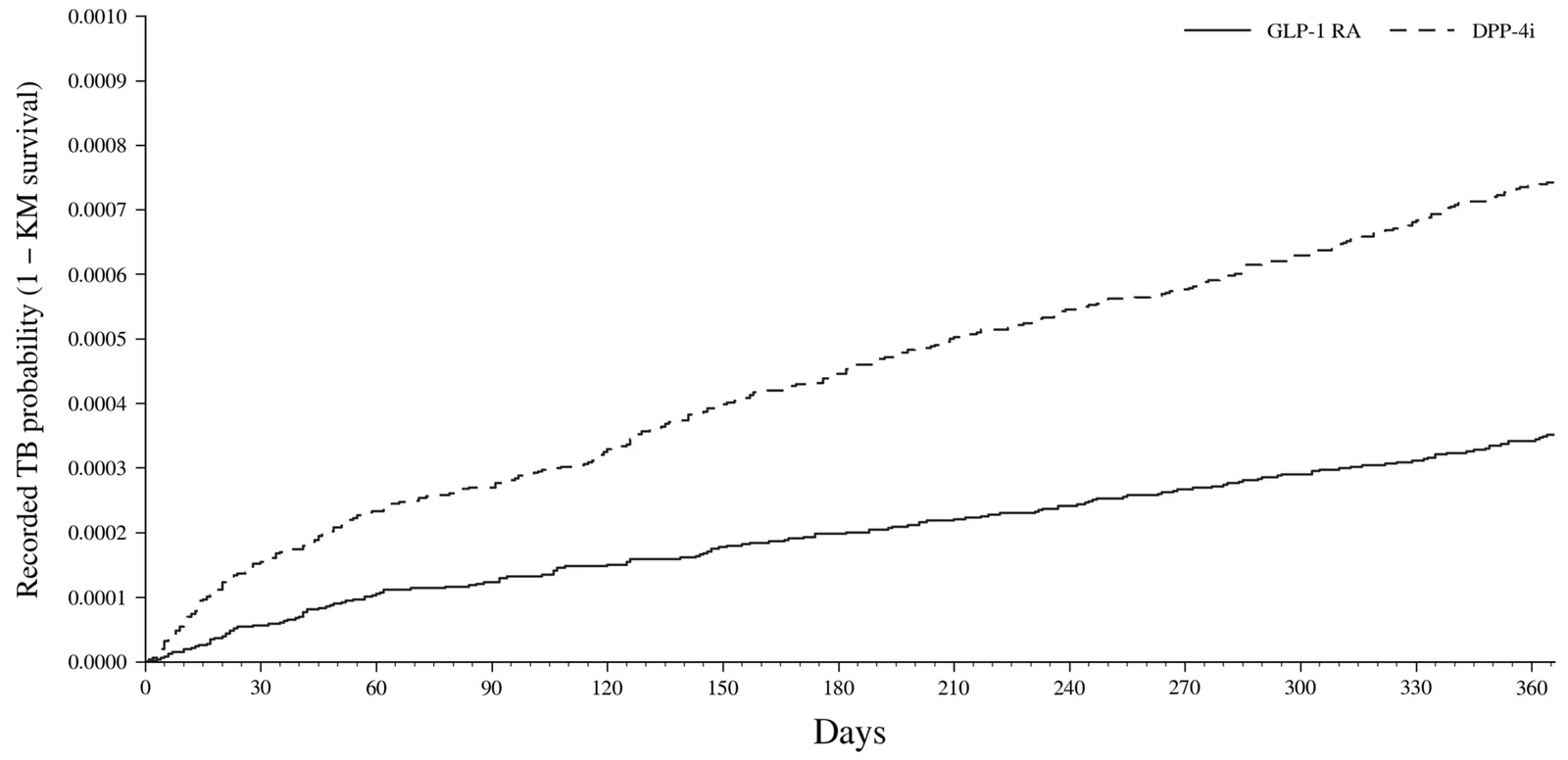
Kaplan–Meier-estimated probability of recorded TB in the GLP-1 RA and DPP-4i cohorts. Curves show 1 minus the Kaplan–Meier survival estimate; death was not modeled as a competing event.

During days 1–365 after the index date, TB was recorded in 155 GLP-1 RA users (0.0332%) and 320 DPP-4i users (0.0685%); the crude absolute risk difference was −0.0353 percentage points (Table 2). The platform-reported hazard ratio (HR; GLP-1 RA vs. DPP-4i) was 0.472 (95% CI, 0.390–0.572). The exploratory subgroup comparisons and the separately matched E11 sensitivity analysis are shown in Figure 2. The Kaplan–Meier-derived curve for recorded TB is shown in Figure 3; its ordinate is 1 minus the Kaplan–Meier estimate and does not represent the cumulative incidence of competing risks. TB event counts for each supplied subgroup are tabulated in Table 3. In the organ-transplant subgroup, there were 23 vs. 42 TB events; the supplied HIV/AIDS subgroup output contained no TB comparison, so a TB HR or TB event count cannot be reported for that stratum. The E11 comparison is a separately matched sensitivity analysis, not a formal interaction test.

### 2.3. Recorded Death

**Figure 4:**
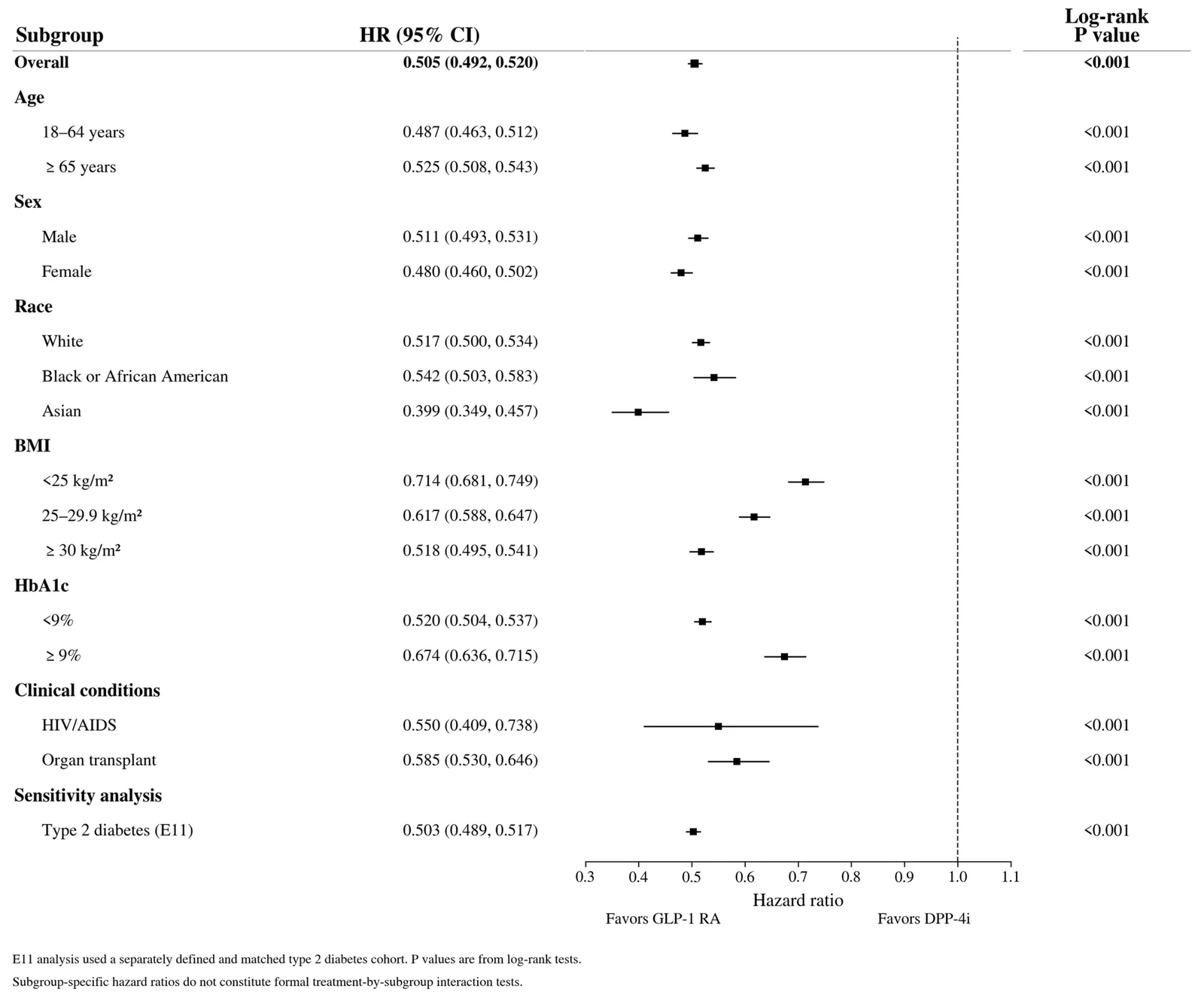
Exploratory subgroup comparisons and E11 sensitivity analysis for the platform-defined death outcome. Squares indicate HRs, and lines show 95% confidence intervals. No formal interaction tests were available.

**Figure 5:**
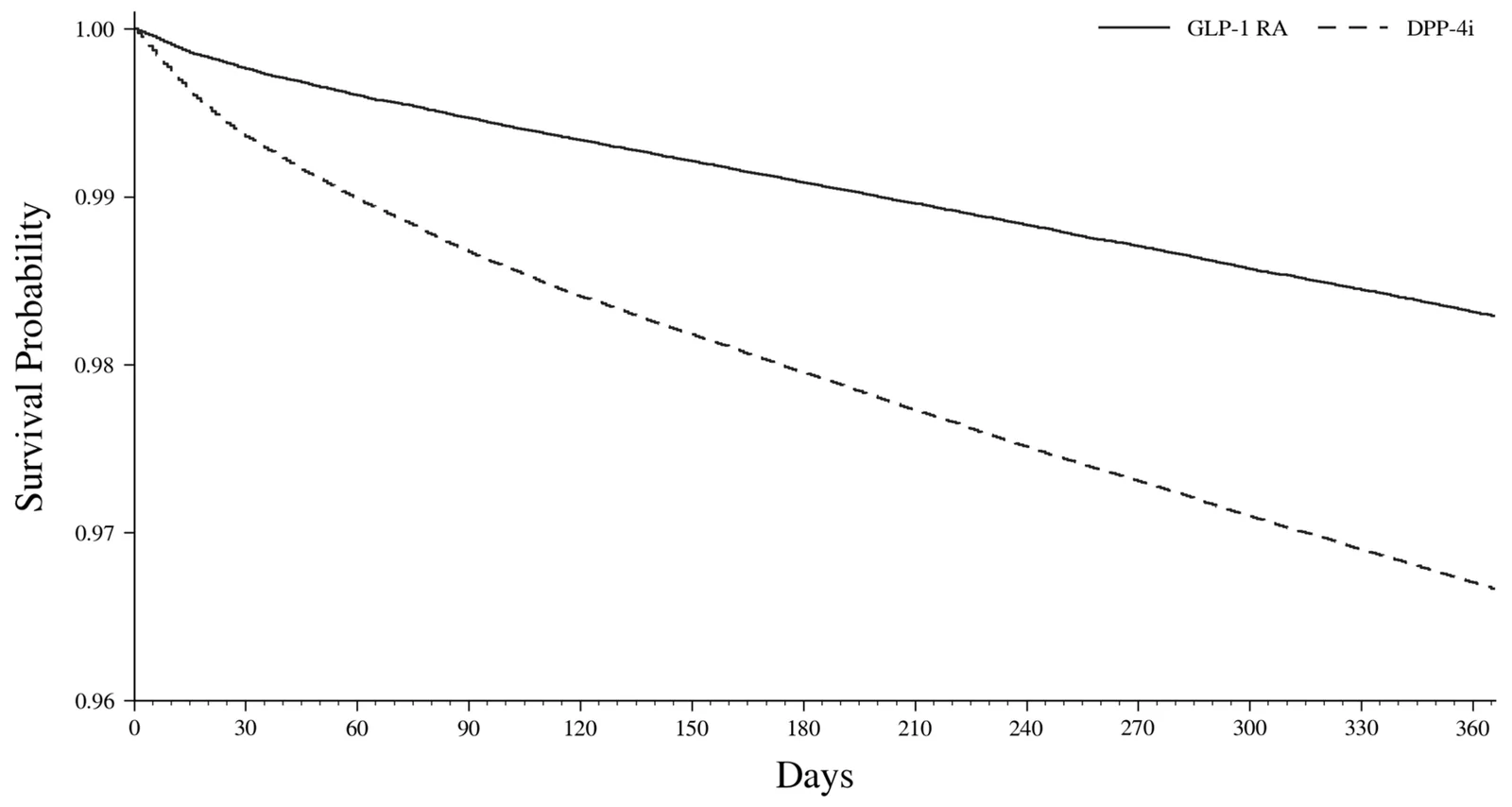
Kaplan–Meier-estimated survival probability for the platform-defined death outcome in the GLP-1 RA and DPP-4i cohorts.

The platform-defined death outcome (deceased demographic indicator or ICD-10-CM R99) was recorded in 7575 GLP-1 RA users (1.6224%) and 14,572 DPP-4i users (3.1210%); the crude absolute risk difference was −1.4986 percentage points. The summary HR was 0.505 (95% CI, 0.492–0.520). Figure 4 shows exploratory subgroup comparisons; Figure 5 shows the Kaplan–Meier survival curves. The platform proportionality test indicated evidence against a constant HR for this outcome, so the single HR should be interpreted with caution.

### 2.4. Gastrointestinal and Hepatobiliary Diagnoses

**Figure 6:**
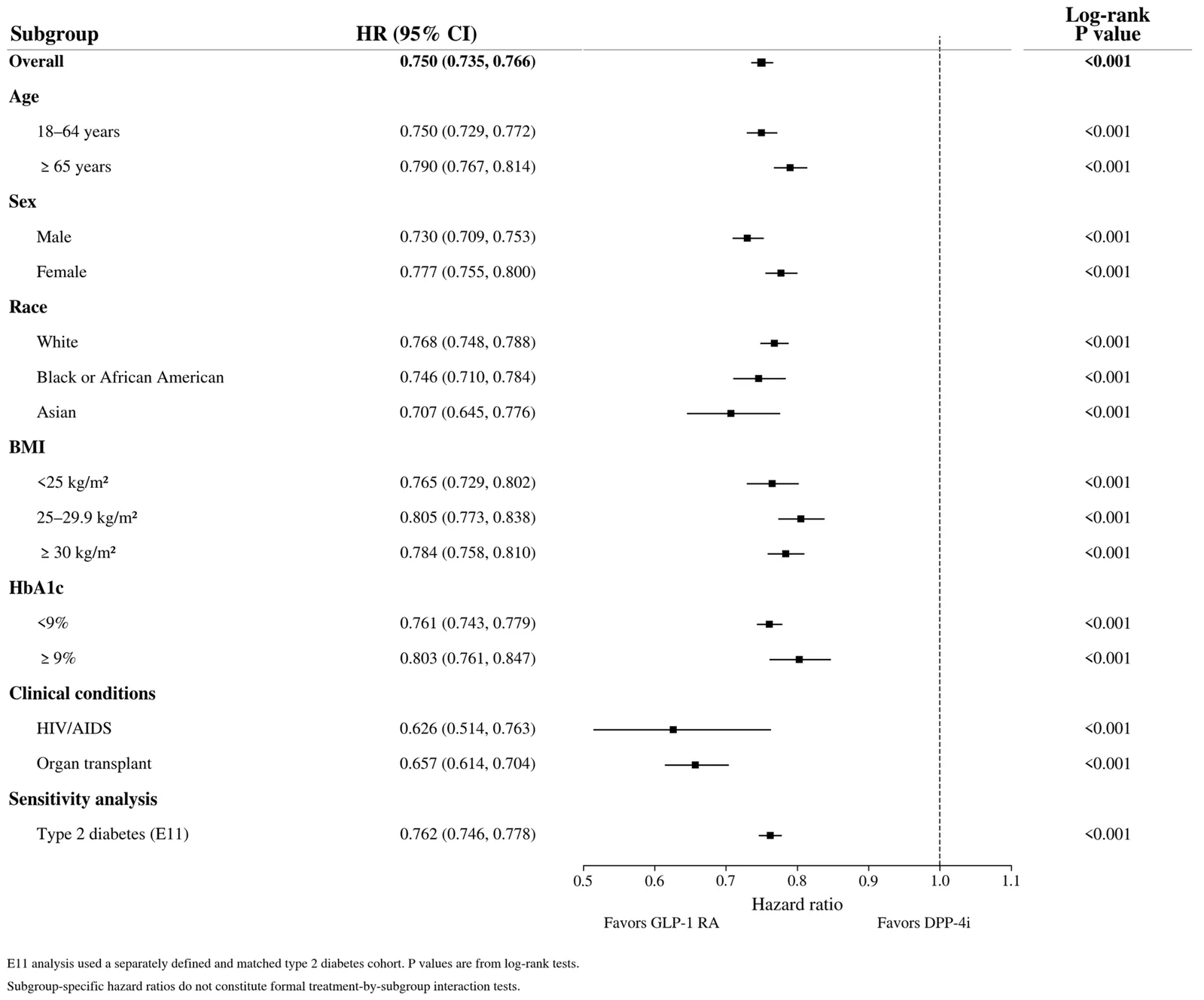
Exploratory subgroup comparisons and E11 sensitivity analysis for recorded gastrointestinal/hepatobiliary diagnoses. Squares indicate HRs, and lines show 95% confidence intervals. No formal interaction tests were available.

**Figure 7:**
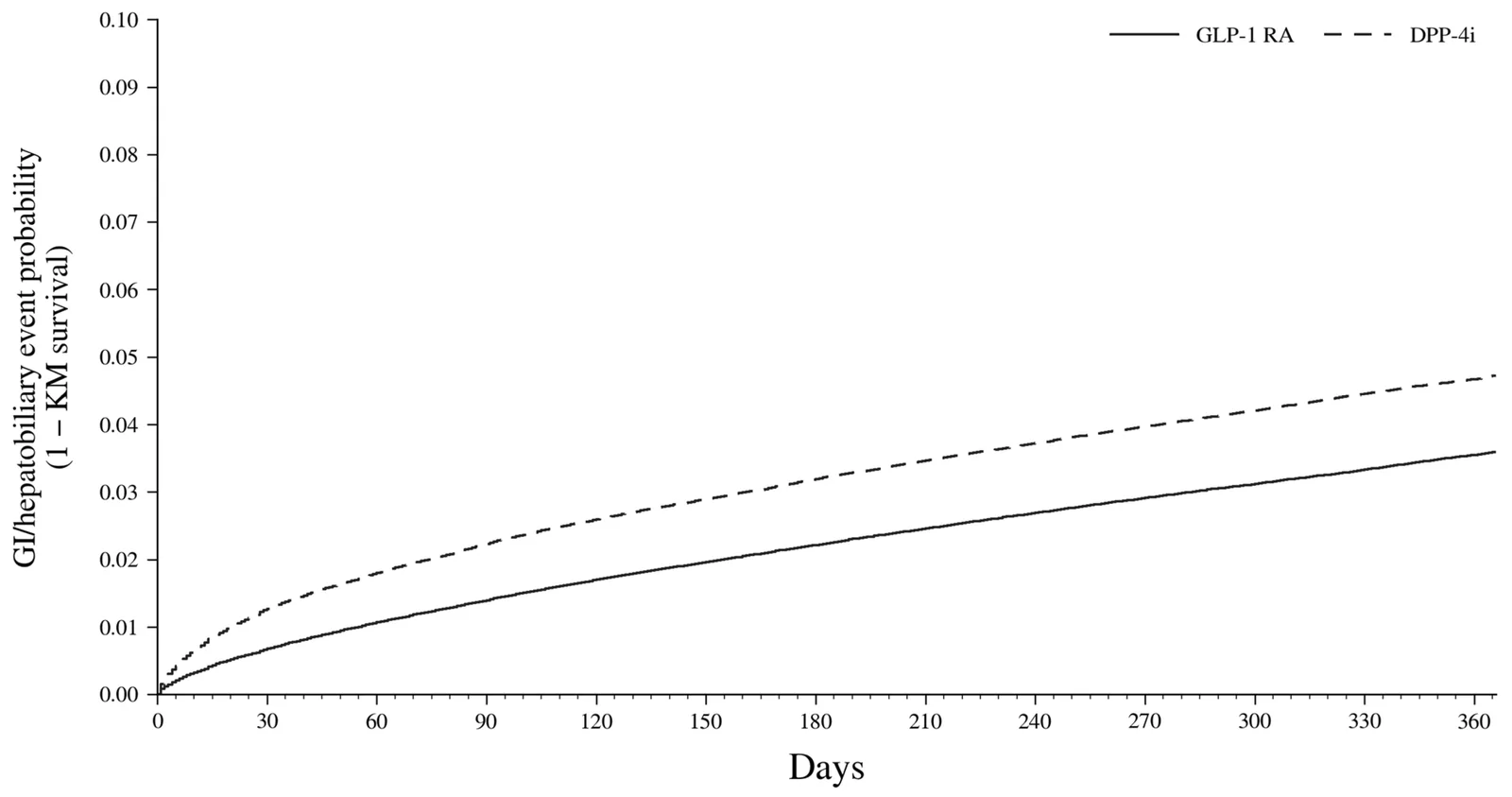
Kaplan–Meier-estimated probability of recorded gastrointestinal or hepatobiliary disorders in the GLP-1 RA and DPP-4i cohorts. Curves show 1 minus the Kaplan–Meier survival estimate; death was not modeled as a competing event.

The composite outcome was recorded in 15,914 GLP-1 RA users (3.4084%) and 20,559 DPP-4i users (4.4033%); the crude absolute risk difference was −0.9949 percentage points. The summary HR was 0.750 (95% CI, 0.735–0.766). Figure 6 presents exploratory subgroup comparisons; Figure 7 shows 1 minus the Kaplan–Meier survival estimates for this composite. The proportionality test indicated evidence against a constant HR, and no competing-risk model for death was fitted.

### 2.5. Type 2 Diabetes Sensitivity and Exploratory Subgroups

The E11-restricted analysis yielded 460,825 matched patients per group. TB was recorded in 159 GLP-1 RA and 309 DPP-4i users (HR, 0.502; 95% CI, 0.414–0.608). The death-definition counts were 7419 and 14,355 (HR, 0.503; 95% CI, 0.489–0.517), and gastrointestinal/hepatobiliary counts were 15,853 and 20,185 (HR, 0.762; 95% CI, 0.746–0.778), respectively. Separate matching was conducted within each reported subgroup, so the event counts are not additive across strata (Supplementary Table S1). For example, the transplant subgroup had 23 versus 42 TB events. The supplied HIV/AIDS output contained no TB comparison; no TB HR or count is reported for that stratum. No formal treatment-by-subgroup interaction test was available; differences across subgroup HRs are not evidence of effect modification.

## 3. Discussion

In this large retrospective cohort study, GLP-1 RA exposure was associated with fewer recorded TB events, deaths, and gastrointestinal/hepatobiliary events than DPP-4i exposure during the first year after cohort entry. These associations were also observed in the separately matched analysis restricted to patients with type 2 diabetes mellitus (E11). However, these findings reflect comparative outcomes observed in routine clinical care and do not establish that GLP-1 RAs prevent TB or reduce mortality.

Several potential mechanisms may help explain the lower hazard of incident TB observed in our study. First, GLP-1 RAs and DPP-4is are both secretin-related drugs with similar mechanisms of action. However, they differ significantly in pharmacological potency and clinical efficacy. Some review studies have shown that GLP-1 RAs provide better glycemic control compared to DPP-4is [26,27,28]. Second, available real-world and mechanistic evidence suggests that GLP-1 RAs may exert immunomodulatory effects distinct from those of DPP-4is. GLP-1 RAs actively regulate immune function through both systemic and central pathways. At the systemic level, they directly activate receptors on various immune cells, restoring the balance between pro-inflammatory Th17 cells and anti-inflammatory regulatory T cells (Tregs) via the PI3K/Akt/FoxO1 signaling pathway [29]. In addition, GLP-1 RAs are involved in the key gut–brain–immune axis, where activation of central neuronal GLP-1 receptors reduces peripheral inflammation, particularly by recruiting central α1-adrenergic and opioid signaling to attenuate the production of TNF-α, IL-1β, and IL-6 induced by multiple Toll-like receptor (TLR) agonists [30].

The observed mortality association was directionally consistent with previous studies reporting lower mortality among GLP-1 RA users than among DPP-4i users in other clinical populations [18,31,32,33,34,35,36,37]. However, our platform-defined mortality outcome combines a deceased indicator with ICD-10-CM code R99, which may capture deaths of unspecified or unknown cause. Evidence against proportional hazards for this outcome means that its summary HR should not be interpreted as constant at every time point. Mortality can also preclude the ascertainment of a subsequent TB or gastrointestinal diagnosis.

The gastrointestinal and hepatobiliary composite event deserves separate interpretation. Our finding that DPP-4i use was associated with a higher incidence of the composite gastrointestinal and hepatobiliary outcome than GLP-1 RA use is broadly consistent with recent pharmacovigilance and real-world epidemiological studies [38,39]. The composite outcome included gastroparesis, gallbladder and bile duct disorders, pancreatitis, and bowel obstruction, which have distinct clinical mechanisms and may be identified through different diagnostic pathways. The lower recorded incidence of the composite outcome among GLP-1 RA users should not be interpreted as evidence of a lower risk for each individual component. Differences in clinical surveillance, underlying disease characteristics, treatment selection, and unmeasured adherence may have contributed to the observed association. Furthermore, the absence of a competing-risk analysis limits the interpretation of this nonfatal outcome.

The strengths of this study include a large, multi-organization cohort, an active drug-class comparator, transparent reporting of event counts, and a separately matched sensitivity analysis restricted to patients with type 2 diabetes mellitus (E11). However, the aggregate TriNetX output does not provide sufficient information to establish the geographic distribution of the analyzed population; therefore, neither a predominantly U.S.-based sample nor multinational representativeness can be assumed from the network name alone. The E11-restricted analysis helps reduce potential heterogeneity arising from the inclusion of diabetes codes other than type 2 diabetes, including O24, but does not eliminate all uncertainties related to diagnostic coding.

Several limitations require emphasis. First, although BMI was included in the propensity score matching, a substantial imbalance remained after matching (absolute standardized mean difference, 0.406). Residual confounding, including confounding by indication—such as differences in baseline characteristics, diabetes severity, comorbidities, concomitant medications, and healthcare utilization—may have influenced the observed outcomes. Second, the prior-year exclusion was applied to the comparator drug class but did not require the absence of prior use of the assigned drug class; therefore, the cohorts cannot be considered confirmed new-user cohorts. Patients were classified according to their index exposure regardless of subsequent changes in treatment. Third, patients with an outcome recorded before the start of the follow-up window were not necessarily excluded from the platform analysis; consequently, outcomes observed during follow-up cannot always be interpreted as first-ever incident events. Fourth, electronic health record coverage may be incomplete, and the platform-defined mortality outcome has not been validated as a cause-specific mortality endpoint. Fifth, the available platform analysis did not provide two-group competing-risk estimates for TB or gastrointestinal/hepatobiliary outcomes, formal tests for subgroup interactions, or observed person-time denominators. Finally, platform-based proportional-hazards testing indicated evidence of nonproportional hazards for mortality and the gastrointestinal/hepatobiliary composite outcome. Collectively, these limitations constrain causal interpretation and may limit the generalizability of the findings, particularly to populations in high-TB-burden settings.

## 4. Materials and Methods

### 4.1. Study Design and Data Source

We conducted a retrospective cohort study using de-identified electronic health records from the TriNetX Global Collaborative Network (TriNetX, Inc., Cambridge, MA, USA). At the time of the analysis, 187 healthcare organizations were queried. The network provides longitudinal records of diagnoses, medications, procedures, laboratory measurements, and recorded mortality. The study was approved by the Institutional Review Board of Kaohsiung Veterans General Hospital (KSVGH25-CT11-13), which waived informed consent for the use of de-identified data. The geographic distribution of the analyzed patients could not be established from the aggregate report.

### 4.2. Cohort Definition and Index Date

We identified adults aged at least 18 years who had a recorded diagnosis of diabetes mellitus (ICD-10-CM E08–E13) or diabetes mellitus in pregnancy, childbirth, or the puerperium (O24) on or before a qualifying medication record between 1 January 2016 and 31 December 2024. The GLP-1 RA cohort was defined by a record of a GLP-1 RA (ATC A10BJ); the active comparator cohort was defined by a record of a DPP-4i (ATC A10BH). The index date was the first qualifying medication event in the specified calendar period for each cohort. For the GLP-1 RA cohort, a recorded DPP-4i exposure in the year before the first GLP-1 RA event was an exclusion criterion; the converse rule was applied to the DPP-4i cohort. Use of the comparator drug after the first exposure was not used to determine cohort eligibility. Patients with a recorded TB diagnosis (A15–A19) or death in the year before the first study-drug event were excluded. Absence of prior use of the assigned study drug was not required; therefore, the cohorts should not be described as confirmed new users. The analyses evaluated patients according to their index cohort and did not reassign exposure when treatment changed after the index date.

### 4.3. Propensity Score Matching

Propensity scores were estimated from recorded baseline demographic, diagnostic, medication, and laboratory characteristics available in the TriNetX analysis, including age, recorded sex, race, comorbidities, concomitant glucose-lowering therapies, glycated hemoglobin, and body mass index (BMI). Patients in the two treatment cohorts were matched 1:1 using the platform propensity score matching procedure. We assessed balance after matching with standardized mean differences and reported any residual imbalance rather than assuming that matching balanced every covariate.

### 4.4. Outcomes and Follow-Up

The primary outcome was a recorded TB diagnosis (ICD-10-CM A15–A19). Secondary outcomes included recorded death, defined in the platform query as either the deceased demographic indicator or ICD-10-CM R99, and a composite of gastrointestinal and hepatobiliary diagnoses: gastroparesis (K31.84), calculus of the bile duct without cholangitis or cholecystitis (K80.5), cholecystitis (K81), cholangitis (K83.0), gallbladder calculus without cholecystitis (K80.2), acute pancreatitis (K85), or ileus or intestinal obstruction without hernia (K56). The first recorded occurrence of each outcome from day 1 through day 365 after the index date was assessed separately. There was no composite outcome combining TB, death, and gastrointestinal or hepatobiliary disorders. The platform analysis included patients with a history of an outcome before the outcome window; accordingly, a recorded follow-up event does not necessarily establish a first lifetime occurrence. For survival analysis, patients without a recorded outcome were censored after the last fact available in their record or at the end of the 365-day window, whichever came first.

### 4.5. Sensitivity and Subgroup Analyses

To evaluate whether inclusion of non–type 2 diabetes diagnoses influenced the findings, an E11-restricted sensitivity analysis was defined by requiring a recorded type 2 diabetes diagnosis (ICD-10-CM E11) on or before the qualifying index medication event in both treatment cohorts. The sensitivity analysis specifies the same treatment-specific index dates, comparator-drug exclusion during the preceding year, outcome definitions, day 1–365 follow-up window, propensity score matching procedure, and outcome comparisons as the main analysis. Exploratory subgroup analyses were defined according to characteristics recorded at baseline, including age, sex, race, BMI, glycated hemoglobin, HIV/AIDS status, and history of organ transplantation, where data and event counts permitted. Each reported subgroup comparison was based on a separately matched cohort; its HR therefore described that subgroup-specific comparison. Formal treatment-by-subgroup interaction tests were not available in the TriNetX analysis interface. Differences in subgroup-specific estimates or their within-subgroup *p*-values were not interpreted as evidence of effect modification.

### 4.6. Statistical Analysis

We summarized baseline characteristics before and after matching and assessed covariate balance using standardized mean differences. For each outcome, the TriNetX Compare Outcomes analysis provided the number of patients with the outcome, the proportion with an outcome during the 365-day window, the between-group risk difference and risk ratio, Kaplan–Meier estimates, a log-rank test, and an HR with a 95% confidence interval. The HR was oriented as GLP-1 RA versus DPP-4i. We report crude absolute one-year risk differences calculated from the displayed event counts. The proportionality tests in the platform output provided evidence against a constant HR over time for the death and gastrointestinal/hepatobiliary outcomes, so the corresponding HRs are interpreted as summary measures. The platform also supplied a test of proportionality; evidence against proportional hazards was considered when interpreting a single summary HR. We did not calculate person-time incidence rates from the available report. For nonfatal outcomes, no competing-risk model accounting for death was available in the analysis interface; thus, the Kaplan–Meier estimates and HRs were interpreted with this limitation. All statistical results were generated within TriNetX; analyses were two-sided, with a nominal *p*-value less than 0.05 used as the threshold for statistical significance.

## 5. Conclusions

This retrospective matched comparative study involving adults with diabetes found that the use of GLP-1 RAs was associated with fewer recorded cases of TB, deaths, and gastrointestinal/hepatobiliary events over a one-year period compared to the use of DPP-4is. A subgroup analysis of patients with E11 (type 2 diabetes) showed similar associations. However, the interpretation of these findings is limited by factors such as residual imbalance in BMI, potential misclassification of exposure, and the absence of a competing-risks analysis. Further research is needed to obtain verified exposure histories and person-time data regarding specific outcomes.

## Figures and Tables

**Table 1:** Baseline characteristics before and after propensity score matching.

Characteristic	Before Matching			After Matching		
	DPP-4i N = 724,752	GLP-1 RA N = 921,454	SMD	DPP-4i N = 466,903	GLP-1 RA N = 466,903	SMD
Age at index, years	63.4 ± 12.2	57.3 ± 12.9	0.484	61.0 ± 12.4	60.7 ± 12.1	0.025
Sex						
Female	342,588 (47.27)	510,879 (55.44)	0.164	233,108 (49.93)	233,197 (49.95)	<0.001
Male	382,164 (52.73)	410,575 (44.56)	0.164	233,795 (50.07)	233,706 (50.05)	<0.001
Race						
White	289,442 (39.94)	604,330 (65.58)	0.532	270,833 (58.01)	265,500 (56.86)	0.023
Black or African American	88,262 (12.18)	168,829 (18.32)	0.172	81,572 (17.47)	78,316 (16.77)	0.019
Asian	84,767 (11.70)	34,580 (3.75)	0.301	27,954 (5.99)	29,491 (6.32)	0.014
Comorbidities						
Essential hypertension	439,810 (60.68)	657,258 (71.33)	0.226	322,366 (69.04)	321,783 (68.92)	0.003
Hyperlipidemia	295,734 (40.80)	496,385 (53.87)	0.264	236,762 (50.71)	237,161 (50.79)	0.002
Diseases of blood and blood-forming organs	178,651 (24.65)	292,843 (31.78)	0.159	140,028 (29.99)	136,530 (29.24)	0.016
Gastroesophageal reflux disease	154,915 (21.37)	300,681 (32.63)	0.256	125,844 (26.95)	127,057 (27.21)	0.006
Ischemic heart diseases	167,961 (23.17)	201,506 (21.87)	0.031	113,129 (24.23)	109,934 (23.55)	0.016
Chronic lower respiratory diseases	126,139 (17.40)	238,789 (25.91)	0.208	99,910 (21.40)	100,667 (21.56)	0.004
Mood disorders	106,747 (14.73)	265,067 (28.77)	0.345	94,987 (20.34)	97,671 (20.92)	0.014
Anxiety and related disorders	111,394 (15.37)	277,767 (30.14)	0.358	95,964 (20.55)	98,925 (21.19)	0.016
Chronic kidney disease	135,020 (18.63)	140,556 (15.25)	0.090	86,756 (18.58)	83,217 (17.82)	0.020
Type 2 DM with neurological complications	97,199 (13.41)	171,606 (18.62)	0.142	80,608 (17.26)	79,873 (17.11)	0.004
Type 2 DM with kidney complications	120,671 (16.65)	146,777 (15.93)	0.020	82,506 (17.67)	79,731 (17.08)	0.016
Psychoactive substance use disorders	83,935 (11.58)	173,337 (18.81)	0.202	73,925 (15.83)	73,744 (15.79)	0.001
Nicotine dependence	67,268 (9.28)	139,301 (15.12)	0.179	59,508 (12.75)	59,414 (12.73)	0.001
Diseases of liver	86,464 (11.93)	151,937 (16.49)	0.131	62,702 (13.43)	63,723 (13.65)	0.006
Cerebrovascular diseases	94,137 (12.99)	90,493 (9.82)	0.100	55,849 (11.96)	54,193 (11.61)	0.011
Type 2 DM with ophthalmic complications	54,804 (7.56)	83,770 (9.09)	0.055	41,312 (8.85)	40,592 (8.69)	0.005
Type 2 DM with circulatory complications	42,213 (5.82)	71,865 (7.80)	0.078	35,326 (7.57)	34,075 (7.30)	0.010
Melanoma and other skin cancers	14,016 (1.93)	24,927 (2.71)	0.051	12,165 (2.61)	11,757 (2.52)	0.006
Malignant neoplasms of breast	14,286 (1.97)	20,240 (2.20)	0.016	10,610 (2.27)	10,214 (2.19)	0.006
Malignant neoplasms of ill-defined	18,612 (2.57)	17,563 (1.91)	0.045	10,946 (2.34)	10,631 (2.28)	0.004
Malignant neoplasms of male genital organs	13,481 (1.86)	14,641 (1.59)	0.021	9406 (2.01)	8905 (1.91)	0.008
Malignant neoplasms of digestive organs	21,925 (3.03)	12,010 (1.30)	0.119	8338 (1.79)	8524 (1.83)	0.003
Malignant neoplasms of lymphoid	10,632 (1.47)	12,209 (1.32)	0.012	7438 (1.59)	7067 (1.51)	0.006
Malignant neoplasms of urinary tract	10,155 (1.40)	9825 (1.07)	0.030	6203 (1.33)	5898 (1.26)	0.006
Unspecified dementia	16,534 (2.28)	6809 (0.74)	0.127	5885 (1.26)	5687 (1.22)	0.004
Malignant neoplasms of female genital organs	6952 (0.96)	10,845 (1.18)	0.021	5190 (1.11)	5085 (1.09)	0.002
Malignant neoplasms of respiratory	9814 (1.35)	5178 (0.56)	0.081	3837 (0.82)	3840 (0.82)	<0.001
Thyroid and other endocrine cancers	4176 (0.58)	6267 (0.68)	0.013	2928 (0.63)	2908 (0.62)	0.001
Human immunodeficiency virus	3172 (0.44)	5596 (0.61)	0.024	2709 (0.58)	2641 (0.57)	0.002
Lip, oral cavity, and pharyngeal cancers	4517 (0.62)	1955 (0.21)	0.064	1341 (0.29)	1472 (0.32)	0.005
Mesothelial and soft tissue cancers	1990 (0.27)	2277 (0.25)	0.005	1353 (0.29)	1277 (0.27)	0.003
Eye, brain, and central nervous system cancers	1867 (0.26)	1637 (0.18)	0.017	1090 (0.23)	1042 (0.22)	0.002
Malignant neuroendocrine tumors	1204 (0.17)	1577 (0.17)	0.001	872 (0.19)	849 (0.18)	0.001
Malignant neoplasms of bone	724 (0.10)	786 (0.09)	0.005	505 (0.11)	497 (0.11)	0.001
Secondary neuroendocrine tumors	431 (0.06)	356 (0.04)	0.009	272 (0.06)	255 (0.05)	0.002
Medications						
Biguanides	320,778 (44.26)	536,607 (58.23)	0.282	242,511 (51.94)	247,990 (53.11)	0.024
Insulin	260,054 (35.88)	404,361 (43.88)	0.164	194,096 (41.57)	193,104 (41.36)	0.004
Sulfonylureas	180,731 (24.94)	232,309 (25.21)	0.006	129,758 (27.79)	128,635 (27.55)	0.005
Sodium-glucose co-transporter 2 inhibitors	64,746 (8.93)	160,198 (17.39)	0.252	55,497 (11.89)	59,872 (12.82)	0.028
Thiazolidinediones	42,686 (5.89)	56,571 (6.14)	0.010	30,812 (6.60)	30,510 (6.53)	0.003
Alpha-glucosidase inhibitors	11,432 (1.58)	4257 (0.46)	0.111	2841 (0.61)	3136 (0.67)	0.008
Laboratory						
Creatinine in serum, plasma or Blood	1.3 ± 3.8	1.1 ± 4.8	0.044	1.2 ± 3.6	1.2 ± 4.4	0.016
Glucose in serum, plasma or blood	175.4 ± 84.0	171.3 ± 79.9	0.050	174.5 ± 79.7	171.8 ± 79.1	0.034
Hemoglobin A1c, %	8.1 ± 2.0	8.1 ± 2.1	0.008	8.1 ± 2.0	8.1 ± 2.0	0.015
BMI, kg/m^2^	31.3 ± 7.5	36.5 ± 8.2	0.658	32.2 ± 7.5	35.3 ± 7.8	0.406
Cholesterol in HDL, mg/dL	43.1 ± 15.9	42.8 ± 15.3	0.022	42.4 ± 16.2	43.0 ± 15.1	0.043
Cholesterol, mg/dL	165.8 ± 49.2	170.4 ± 48.5	0.094	165.9 ± 50.3	166.6 ± 48.3	0.013
Cholesterol in LDL, mg/dL	90.4 ± 38.1	92.2 ± 38.3	0.050	89.6 ± 38.7	89.2 ± 37.9	0.010
Triglyceride, mg/dL	174.9 ± 166.5	186.6 ± 183.3	0.067	179.4 ± 170.0	181.0 ± 173.4	0.009

Values are n (%) or mean ± SD. SMD is the absolute standardized mean difference. BMI remained imbalanced after matching (SMD, 0.406). Laboratory summaries reflect available data; GLP-1 RA, glucagon-like peptide-1 receptor agonist; DPP-4i, dipeptidyl peptidase-4 inhibitor.

**Table 2:** Outcome summary of results.

Outcome	GLP-1 RA Events	DPP-4i Events	Crude Risk Difference *	HR (95% CI)
Tuberculosis event	155	320	−0.0353 pp	0.472 (0.390–0.572)
Death definition	7575	14,572	−1.4986 pp	0.505 (0.492–0.520)
GI/hepatobiliary disorders	15,914	20,559	−0.9949 pp	0.750 (0.735–0.766)

* Percentage points, calculated from displayed event counts divided by 466,903 in each group; the platform rounds its risk differences to three decimal places. These are crude one-year proportions, not competing-risk estimates. HR, hazard ratio.

**Table 3:** TB event counts in separately matched strata.

Stratum	GLP-1 RA TB	DPP-4i TB
Age 18–64 years	73	201
Age ≥ 65 years	80	123
Male	70	183
Female	84	143
White	49	96
Black or African American	35	35
Asian	35	67
BMI < 25 kg/m^2^	45	85
BMI 25–29.9 kg/m^2^	51	70
BMI ≥ 30 kg/m^2^	52	85
HbA1c < 9%	124	252
HbA1c ≥ 9%	27	45
HIV/AIDS	Not reported	Not reported
Organ transplantation	23	42
Type 2 diabetes E11 †	159	309

Subgroups were separately matched, and their counts are not additive. † E11 was a separate sensitivity analysis. The HIV/AIDS output provided death and gastrointestinal/hepatobiliary outcomes but no TB outcome.

## Data Availability

Data may be obtained from a third party and are not publicly available. The data that support the findings of this study were obtained from the TriNetX Research Network. Restrictions apply to the availability of these data, which were used under license for the current study and therefore are not publicly available. Data may be available from TriNetX upon reasonable request and with permission from TriNetX. All data provided by TriNetX were de-identified in compliance with applicable privacy regulations.

## Supplementary Materials

The following supporting information can be downloaded at: https://www.mdpi.com/article/10.3390/ijms27209137/s1.
