# Supplementary material for: Association of Glucagon-like Peptide-1 Receptor Agonists Versus Dipeptidyl Peptidase-4 Inhibitors with Tuberculosis and Mortality in Adults with Diabetes
Source: Int J Mol Sci. 2026 Oct 15;27(20):9137. doi: 10.3390/ijms27209137 (PMC13705376; doi:10.3390/ijms27209137)
Supplement: Supplementary file 1 [file ijms-27-09137-s001.zip › ijms-4576979-supplementary.pdf]

Supplementary Table S1. Tuberculosis events in exploratory subgroups and the E11 sensitivity analysis

Subgroup	Matched patients per arm	GLP-1 RA TB events	DPP-4i TB events
Age 18–64 years	263,648	73	201
Age ≥65 years	198,266	80	123
Male	236,010	70	183
Female	217,342	84	143
White	270,356	49	96
Black or African American	80,580	35	35
Asian	26,571	35	67
BMI <25 kg/m²	57,833	45	85
BMI 25–29.9 kg/m²	110,071	51	70
BMI ≥30 kg/m²	185,893	52	85
HbA1c <9%	325,594	124	252
HbA1c ≥9%	68,700	27	45
HIV/AIDS	3,859	Not reported	Not reported
Organ transplantation	15,609	23	42
Type 2 diabetes E11	460,825	159	309

Subgroup analyses were matched separately; event counts across strata are not additive. The E11 comparison is a separately matched sensitivity analysis. The supplied HIV/AIDS output did not include a tuberculosis comparison, so the tuberculosis events and hazard ratio cannot be reported for this stratum.
